# miR-212 promotes pancreatic cancer cell growth and invasion by targeting the hedgehog signaling pathway receptor patched-1

**DOI:** 10.1186/1756-9966-33-54

**Published:** 2014-06-25

**Authors:** Chenchao Ma, Kate Nong, Bo Wu, Bo Dong, Yueqing Bai, Hongda Zhu, Weiwei Wang, Xinyu Huang, Zhou Yuan, Kaixing Ai

**Affiliations:** 1 Department of General Surgery, The Sixth People’s Hospital Affiliated to Shanghai Jiaotong University, Shanghai 200233, China; 2 Department of Pathology, The Sixth People’s Hospital Affiliated to Shanghai Jiaotong University, Shanghai 200233, China

**Keywords:** miR-212, Patched-1, Pancreatic cancer, Proliferation, Migration, Invasion

## Abstract

**Background:**

microRNAs (miRNAs) are a class of small non-coding RNAs that play important roles in carcinogenesis. In the present study, we investigated the effect of miR-212 on pancreatic ductal adenocarcinoma (PDAC) and its target protein.

**Methods:**

Quantitative real-time PCR(qRT-PCR) was performed to detect the expression of miR-212 in PDAC tissues and pancreatic cancer cell lines. miR-212 mimic, miR-212 inhibitor and negative control were transfected into pancreatic cancer cells and the effect of miR-212 up-regulation and down-regulation on the proliferation, migration and invasion of cells were investigated. Furthermore, the mRNA and protein levels of Patched-1(PTCH1) were measured. Meanwhile, luciferase assays were performed to validate PTCH1 as miR-212 target in PDAC.

**Results:**

miR-212 was up-regulated in PDAC tissues and cells.Using both gain-of function and loss-of function experiments, a pro-oncogenic function of miR-212 was demonstrated in PDAC. Moreover, up-regulated of PTCH1 could attenuate the effect induced by miR-212.

**Conclusion:**

These data suggest that miR-212 could facilitate PDAC progression and metastasis through targeting PTCH1, implicating a novel mechanism for the progression of PDAC.

## Introduction

microRNAs (miRNAs) constitute an evolutionarily conserved class of small, non-coding RNAs whose function are regulating expression of multiple genes either by translational suppression or gene degradation via interactions with 3′untranslated regions (UTR) of target mRNAs [1]. It has been confirmed that miRNAs modulate many key cellular processes, such as cell growth, cycle, differentiation, and cell death [2,3]. Moreover, Dysregulation of miRNA expression has been identified in various types of cancer, and compelling evidence suggest that miRNAs function as oncogenes or tumor suppressors genes [4,5]. Recently, miRNAs have been discovered to have a role in progression and metastasis of human cancers [6-11]. Furthermore, several clinical studies have observed correlations between miRNA expression and recurrence and survival [12]. The miR-212, which locates at chromosome 17p13.3, was shown to be over-expressed in many cancers, including non-small cell lung cancer [13], and oral carcinoma [14], whereas in other tumors such as hepatocellular carcinoma [15], gastric cancer [16,17], colorectal cancer [18], prostate cancer [19], miR-212 was down-expressed. In the present study, we aimed at exploring the roles miR-212 played in PDAC and the potential mechanism. Using gain and loss of function assays, a pro-oncogenic function of miR-212 in PDAC was observed. Furthermore, the Hh signal pathway receptor PTCH1 was demonstrated to be a direct target of miR-212 in PDAC. Our findings provide an oncogenic role of miR-212 orchestrates in PDAC and its possible mechanism.

## Materials and methods

### Ethical approval of the study protocol

For the analyzed tissue specimens, all patients were given informed consent to use excess pathological specimens for research purposes. The protocols employed in this study and the use of human tissues was approved by the Ethics Committee of The Sixth People’s Hospital affiliated of Shanghai Jiaotong University and conducted in full accordance with ethical principles, including the World Medical Association Declaration of Helsinki, and the local legislation. The manuscript was accompanied by a statement that the experiments were undertaken with the understanding and written consent of each subject and according to the above mentioned principles.

### Human tissue specimens and cell lines

Twenty-two PDAC specimens were obtained from The Sixth People’s Hospital affiliated to Shanghai Jiaotong University. The matched normal gastric tissue samples were obtained from tissues that were located 5 cm away from the tumor margin. The study was approved by the Ethics Committee of The Sixth People’s Hospital affiliated of Shanghai Jiaotong University.

The human pancreatic cancer cells, including PANC-1, SW1990, BxPC-3 were obtained from the Americacn Type culture Collection (ATCC, manassas, VA) and the normal human pancreatic duct epithelial cells were isolated from normal pancreatic tissues as previously described [20]. Cells were maintained in DMEM with 10% FBS (GIBCO, Carlsbad, CA), and were cultured at 37°C with 5% CO_2_.

### Quantitative real-time PCR(qRT-PCR)

Total RNA was extracted from tissues and cells using Trizol regent (Invitrogen, Carlsbad, CA), and the reverse transcription reactions were performed by random primers and a Moloney murine leukemia virus reverse transcriptase kit (Invitrogen) following the manufacturer’s protocol. Real-time PCR was performed using a standard SYBR Green PCR kit (Toyobo, Osaka, Japan) protocol on Applied Biosystems 7500 Real Time PCR system (Applied Biosystems, Foster City, CA) according to the instructions. U6 was used as references for miR-212, GAPDH was used as references for PTCH1. Each sample was analyzed in triplicate. The 2^-ΔΔCt^ method was used to quantify the relative levels of gene expression. The primer sequences were as follows: RT Primer: CTCAACTGGTGTCGTGGAGTCGGCAATTCAGTTGAGGGCCGTGA, has-miR-212 forward: ACACTCCAGCTGGGTAACAGTCTCCAGTC; U6 SLRT: CTCAACTGGTGTCGTGGAGTCGGCAATTCAGTTGAGAAAATATG, U6 forward: ACACTCCAGCTGGGCGCAAATTCGTGAAGC. PTCH1 forward: GCTTCCCGTGCTTTTGTCTT, reverse: CTGCAGCTCAATGACTT.

### Transient transfection

miR-212 mimic, miR-212 inhibitor, negative control were obtained from GenePharma (Shanghai, China) and the sequences were as follows: miR-212 mimic: sense (5′ to 3′) UAACAGUCUCCAGUCACGGCC, antisense (5′ to 3′) CCGUGACUGGAGACUGUUAUU. miR-212 Inhibitor: sense (5′ to 3′) GGCCGUGACUGGAGACUGUUA. Negative control: sense (5′ to 3′)UUCUCCGAACGUGUCACGUTT, antisense (5′ to 3′)ACGUGACACGUUCGGAGAATT. Cells were seeded in 6-well plates at a concentration of 1 × 10^5^ and cultured in medium without antibiotics for approximately 24 h before transfection. Cells were transiently transfected with miR-212 mimic, miR-212 inhibitor or negative control(NC), at a final concentration of 200nM. After 6 h incubation at 37°C and 5% CO_2_, the medium was re-placed with fresh culture medium.

### Cell proliferation assay

Cell proliferation analysis was performed with Cell Counting Kit-8 (Dojindo, Kumamoto, Japan) according to the manual of the manufacturer. Briefly, PDAC cells were plated in 96-well plates in triplicate at 1 × 10^4^ cells each well and cultured in the growth medium. Cells were examined at 24, 48, 72, and 96 h. CCK-8 (10 μl) was added to each well at different time points. After an incubation of 1 h at 37°C, absorbance was measured at 450 nm. Five independent experiments were performed.

### Colony formation assay

2000 of each transfected cells were plated in six-well plate and cultured for 14 days. Then cells were fixed and stained with methanol for 30 min, followed by 0.5% crystal violet for 20 min. Visible colonies were quantified in four different fields and the mean value was calculated.

### Cell migration and invasion assays

The wound healing assay was used to examine cell migration. The migration status was determined by measuring the movement of cells into a scraped area created by a 200 μl pipette tip. After wound scratching, cells were cultured in media supplemented with 0.1% FBS to eliminate the effect of cell proliferation. The process of wound closure was photographed at 24 h. Cell invasion was examined using a extracellular matrix membrane (BD Biosciences, San Jose, CA). Cells were suspended in serum-free medium and placed in the top chambers, and complete medium containing 10% FBS was added to the bottom chambers. The chambers were then incubated for 12 h at 37°C with 5% CO_2_. After incubation, the noninvasive cells were gently removed from the top wells with a cotton-tipped swab and the chambers were fixed with methanol for 30 min. The chambers were then stained with crystal violet for another 30 min. Four random fields were counted per chamber using an inverted microscope (Olympus, Japan), and each experiment was repeated three times.

### Luciferase activity assay

PANC-1 cells were co-transfected with miR-212 mimic or negative control (NC) and wild type (WT) or the mutated 3′UTR (Mut) of PTCH1. 48 h later, cells were collected and luciferase activity was assayed using dual-luciferase assay system (Promega, Wiscosin, WI).

### Western blot analysis

Cells were washed with PBS and lysed for 10 min on ice in RIPA buffer (Thermo Scientific, Waltham, MA). Protein concentration was measured using the BCA assay (Thermo Scientific, Waltham, MA). The protein fractions were resuspended in loading buffer and denatured at 100°C for 10 min. Total proteins (20 μg/lane) were separated on 10% SDS polyacrylamide gels and transferred to PVDF membranes. The membranes were then blocked in 5% fat-free milk in TBST buffer (0.1% Tween-20) for 2 h at room temperature. The rabbit anti-human polyclonal antibody was used in conjunction with 0.4 μg/ml of anti-species conjugated horseradish peroxidase (Upstate, Lake Placid, NY), and bands were detected by chemiluminescence (Amersham Pharmacia Inc, Piscataway, NJ).

### Immunohistochemistry

PTCH1 was detected in paraffin-embedded tumor tissues and adjacent normal tissues. Immunohistochemistry staining was performed according to the manufacturer instructions. each slide was deparaffinized in 60°C, followed by treatment with xylene and graded alcohol. After the antigen retrieval and being blocked with 5% bovine serum albumin, tissue slides were immunohistochemically stained by antibody against PTCH1 (Santa Cruz Biotech, Santa Cruz, CA), then visualized by standard avidin–biotinylated peroxidase complex method. Hematoxylin was used for counterstaining and morphologic images were observed with Olympus BX51 microscope.

### Statistical analysis

All data are expressed as mean **±** standard error of mean (SEM), Values of P < 0.05 were considered statistically significant. Statistical analyses were analyzed using Student’s t-test. All analyses were performed with SPSS 19.0.

## Results

### Upregulated miR-212 expression in PDAC tissues and cell lines

**Figure 1:**
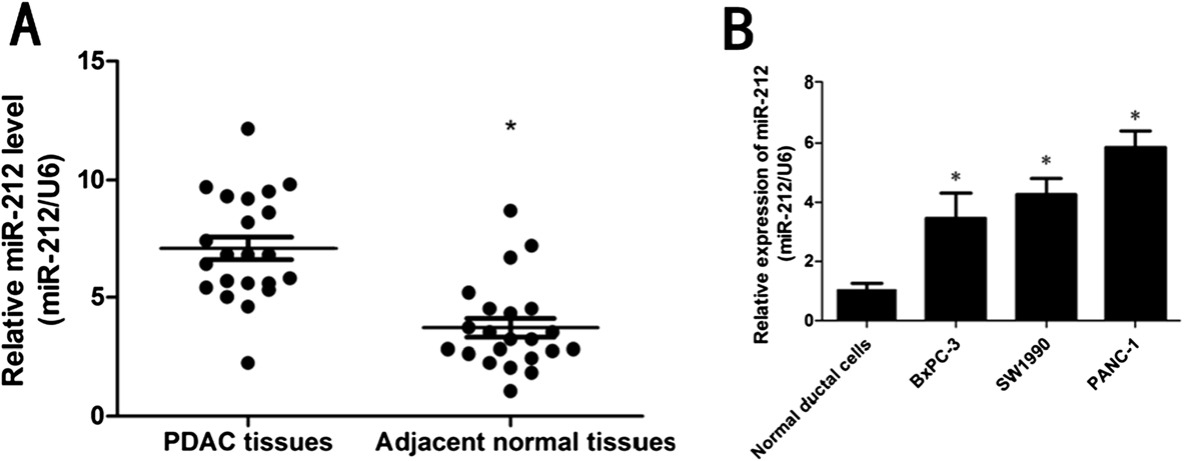
**miR-212 was up-regulated in pancreatic ductal adenocarcinoma (PDAC) tissues and cell lines. (A)** The expression levels of miR-212 were examined by qRT-PCR in PDAC tissues and paired adjacent normal tissues of twenty-two patients. Transcript levels were normalized to U6 expression. **(B)** qRT-PCR analysis of relative miR-212 expression in five PDAC cell lines and normal pancreatic ductal epithelial cells. Transcript levels were normalized to U6 expression. Date are shown as mean ± SEM of three independent experiments. *P < 0.05.

We firstly tested the expression of miR-212 in PDAC tissues by qRT-PCR from 22 patients who underwent pancreaticoduodenectomy. The results verified that the expression of miR-212 was higher in PDAC tissues compared with adjacent normal tissues (Figure 1A). We then examined the expression level of miR-212 in PDAC cell lines (PANC-1, SW1990, BxPC-3). Compared to normal human pancreatic duct epithelial cells, miR-212 expression was significantly upregulated in cancer cell lines (Figure 1B).

### miR-212 increased PDAC cell growth and motility in vitro

**Figure 2:**
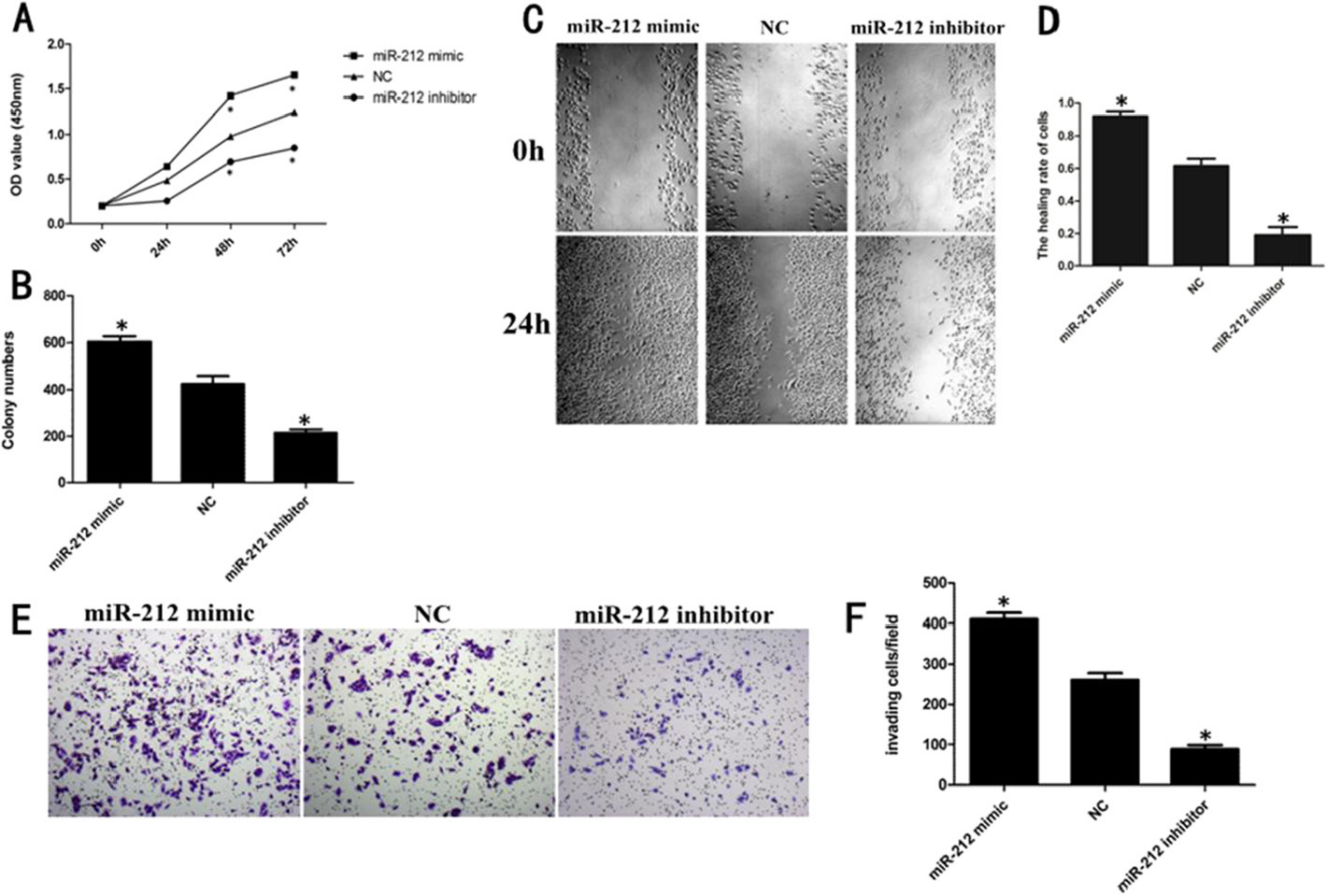
**miR-212 promoted PDAC cell proliferation and motility. (A)** CCK-8 assays performed to examine PANC-1 cells proliferation. Transfection of miR-212 mimic significantly increased PANC-1 cells proliferation, while transfection of miR-212 inhibitor significantly decreased PANC-1 cells proliferation. **(B)** Colony formation assay showed that miR-212 mimic increased PANC-1 cells colony formation, while miR-212 inhibitor significantly inhibited cell colony formation. **(C)** The wound healing assays showed the migration ability of PANC-1 cells transfected with miR-212 mimic or miR-212 inhibitor, statistical analysis is shown in **(D)**. **(E)** Transwell assays showed that miR-212 significantly enhanced PANC-1 cell invasion, while miR-212 inhibitor weaken cell invasion. statistical analysis is shown in **(F)**. Three independent experiments were performed. *P < 0.05.

PANC-1 cells were transfected with miR-212 mimic or miR-212 inhibitor or NC. To explore whether miR-212 have an effect on PDAC cell proliferation, CCK-8 assays were measured. The viability of the miR-212 mimic group was higher compared with NC group, whereas, the miR-212 inhibitor group was lower than NC group (Figure 2A). Similarly, Forced expression of miR-212 significantly increased colony formation of PANC-1 cells (Figure 2B).To test the metastatic ability of miR-212, the wound healing assays were performed in vitro, and it turned out that miR-212 significantly enhanced PANC-1 cell migration capabilities (Figure 2C,D). Similar results were observed in cell invasion assays (Figure 2E,F).

### PTCH1 was a target of miR-212 in PDAC cells

**Figure 3:**
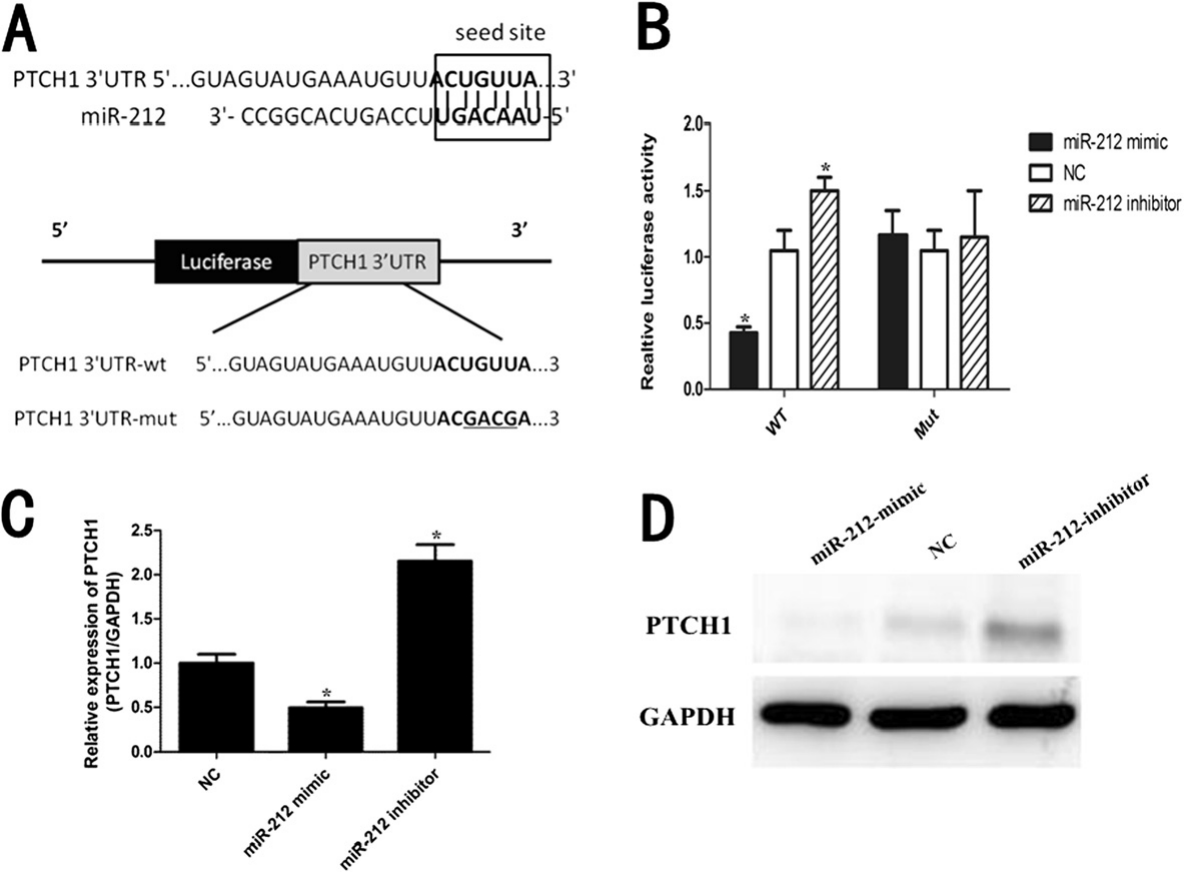
**PTCH1 was a direct target of miR-212 in PDAC cells. (A)** The miR-212 biding site predicted in the 3′UTR of PTCH1 mRNA. Mutant was generated at the seed region of PTCH1 3′UTR as indicated by the underline. A 3′UTR fragment of PTCH1 mRNA containing wild-type (WT) or mutant (Mut) of the miR-212 binding sequence was cloned into the downstream of the luciferase gene in pGL3b vector. **(B)** PANC-1 cells were transfected with pGL3b reporter vectors containing either wild-type (WT) or mutant (Mut) PTCH1 3′UTR with either miR-212 mimic or miR-212 inhibitor, or negative control (NC). Luciferase activity was determined 48 h after transfection. **(C)** PTCH1 mRNA was detected by qRT-PCR in PANC-1 cells transfected with miR-212 mimic or NC or miR-212 inhibitor. **(D)** The protein levels of PTHC1 were examined by western blot assays transfected with miR-212 mimic or miR-212 inhibitor or NC. *P < 0.05.

Targetscan software ( http://www.targetscan.org/) showed that the 3′UTR of human PTCH1 contains putative target sites for miR-212 (Figure 3A). To confirm the target sites, the 3′UTR and mutated 3′UTR of human PTCH1 were amplified and inserted into pGL3b vector. Luciferase activity assay found that miR-212 mimic significantly suppressed the WT but not the Mut 3′UTR luciferase activity in PANC-1 cells (Figure 3B). Furthermore, overexpression of miR-212 significantly inhibited PTCH1 mRNA and protein levels in PANC-1 cell, while inhibition of miR-212 showed opposite effects (Figure 3C,D).

### PTCH1 was negatively correlated with miR-212 in PDAC tissues

**Figure 4:**
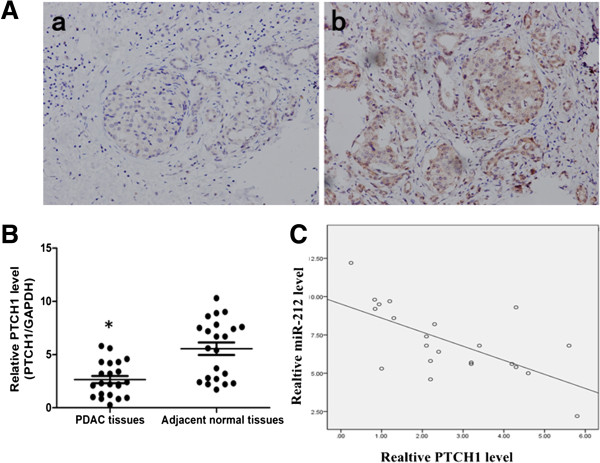
**PTCH1 was negatively correlated with miR-212 in PDAC tissues. (A)** Immunostaining of PTCH1 in PDAC tissues. a. Immunostaining of PTCH1was very weekly positive in PDAC tissues. b. PTCH1was positive in adjacent normal tissues. **(B)** PTCH1 mRNA level was examined by qRT-PCR and it was remarkably decreased in PDAC tissues. **(C)** PTCH1 mRNA level was inversely correlated with miR-212 level in PDAC tissues. (Spearman’s correlation analysis,r = -0.5157, P = 0.001). *P < 0.05.

Next, we detected PTCH1 in PDAC tissues and corresponding non-tumor tissues. Immunostaining showed that 18 of 22(81%) adjacent normal tissues were positive for PTCH1 (Figure 4A,b). In contrast, 16 of 22(72%) PDAC tissues showed less immunostaining (Figure 4A,a). We also found that PTCH1 mRNA was significantly decreased in PDAC tissues compared with corresponding normal tissues (Figure 4B). Moreover, PTCH1 mRNA level was inversely correlated with miR-212 level in PDAC tissues (Figure 4C).

### Overexpression of PTCH1 attenuated miR-212 induced PDAC cell proliferation, migration, and invasion

**Figure 5:**
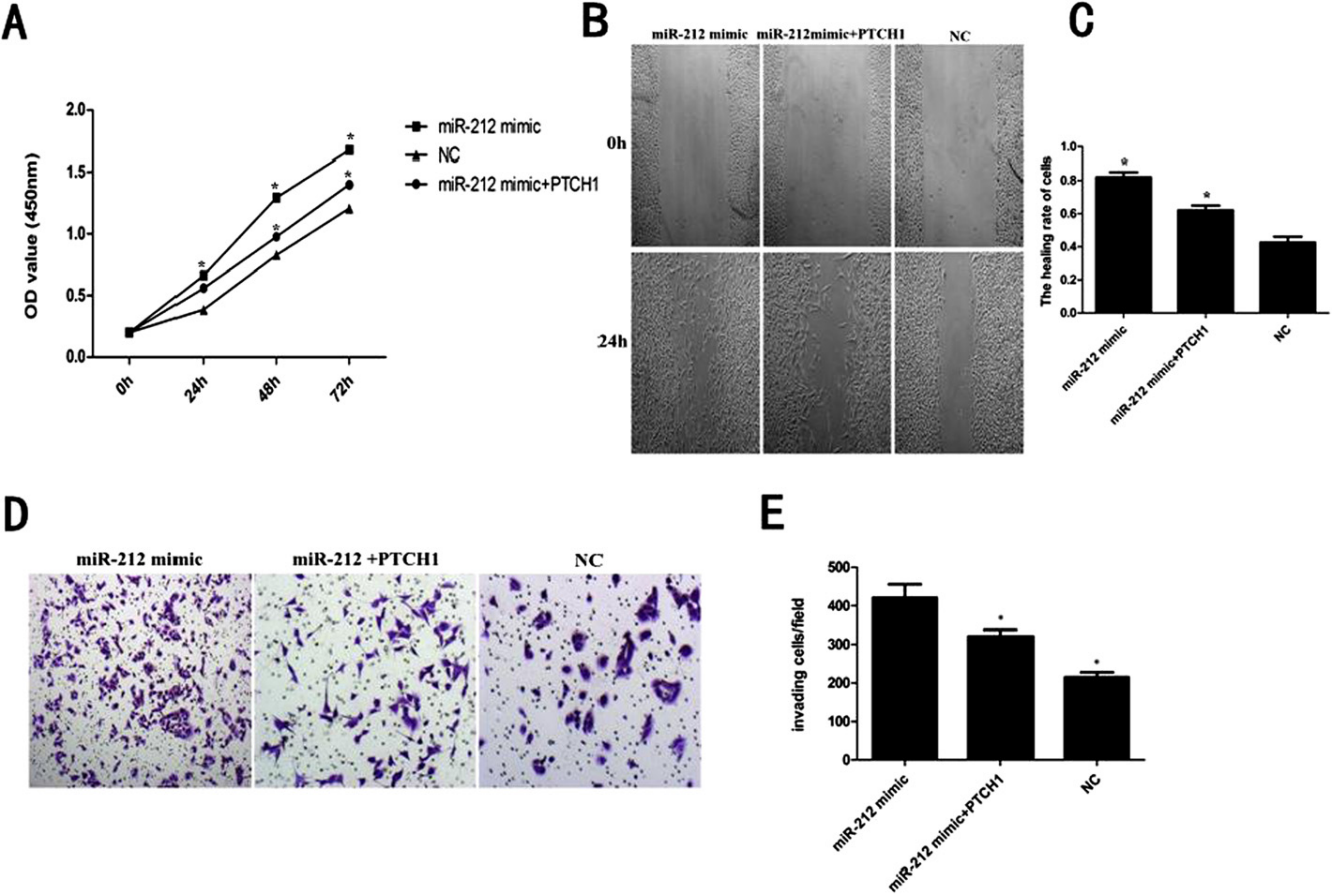
**PTCH1 contributes to miR-212 induced proliferation, migration, invasion of PDAC cells. (A)** PANC-1 cells were co-transfected with miR-212 mimic and PTCH1 or miR-212 mimic or NC, CCK-8 assay was used to examine proliferation. **(B)** The wound healing assay was used to assay migration of PANC-1 cells transfected with miR-212 mimic and PTCH1 or miR-212 mimic, statistical analysis is shown in **(C)**. **(D)** The invasion assay showed invasion capability of PANC-1 cells tranfected with miR-212 mimic and PTCH1 or miR-212 mimic, statistical analysis is shown in **(E)**. *P < 0.05.

To investigate whether overexpression could reverse miR-212 induced cell proliferation, migration, and invasion. PANC-1 cells were co-transfected with miR-212 mimic and PTCH1 or miR-212 mimic or NC. Cell proliferation assay (Figure 5A), in vitro migration (Figure 5B,C) and invasion assays (Figure 5D,E) showed that overexpression of PTCH1 significantly attenuated miR-212 induced PDAC cell proliferation, migration, and invasion.

## Discussion

Accumulating evidences have revealed that miRNAs participate in the progression of many types of cancer by targeting multiple genes which involved in the progression and metastasis [21]. Thereby, identification of specific miRNAs may provide clues for the diagnosis and therapy of patients with cancer. Here, we found that the expression of miR-212 was significantly up-regulated in PDAC tissues and cells lines, consistent with the study of Park [22]. Next, in vitro experiments were performed to test the pro-oncogenic roles of miR-212 played in PDAC. As we expected, a pro-proliferative, pro-migratory, pro-invasive effect of miR-212 was observed in PDAC cells, while miR-212 inhibitor suppressed this effect. Furthermore, we showed that PTCH1 was a direct target of miR-212 in PDAC cells, consistent with Li study, PTCH1 was a direct target of miR-212 in non-small lung cancer [13].

PTCH1 is a member of the hedgehog (Hh) signaling pathway [23]. The Hh signaling pathway is crucial in the growth and patterning during embryonic development, while abnormal activation of Hh signaling pathway is highly involved in tumor progression and metastasis [24,25]. As a tumor suppressor gene, PTCH1 inhibits tumor cell growth and motility by blocking the Hh signaling pathway [26]. PTCH1 is down-regulated in some malignant cancers, such as liver, breast, and esophageal cancer [27-29]. The ectopic expression of PTCH1 may leads to the loss of its normal inhibitory function on Smo, another member of Hh signaling pathway, resulting in the abnormal activation of the Hh signaling pathway, as well as the downstream transcription factor Gli1. The abnormal activation of Hh signaling pathway can lead to the promotion of tumor cell proliferation, migration, and invasion [30-32]. In this study, we showed that the expression level of miR-212 was inversely correlated with PTCH1 in PDAC tissues, and up-regulated PTCH1 could attenuate the pro-oncogenic effect induced by miR-212 in PDAC cells. These results indicated that the overexpression of miR-212 mediated PDAC cell growth, migration, and invasion may partially through inhibiting PTCH1 expression.

## Conclusions

Taken together, this study showed that miR-212 was up-regulated in PDAC samples and cell lines, and both gain-of and loss of-function demonstrated that miR-212 enhanced cell proliferation, colony formation, migration, and invasion of PDAC cells. PTCH1 was identified as a target of miR-212, and up-regulated PTCH1 partially attenuated the pro-oncogenic effect or miR-212, indicating that miR-212 may act as an oncogene and miR-212/PTCH1 present a potential target for PDAC therapy.

## Competing interests

The authors declare that they have no competing interests.

## Authors’ contributions

CM, KN, BW, BD, YB, XH performed most of the experiments. KA designed the study. HZ, ZY and WW performed statistical analysis. KA supervised the study, and CM wrote the manuscript. All authors read and approved the final manuscript.
